# Intemperance as a Disease

**Published:** 1869-11

**Authors:** 


### Society Reports.

ARTICLE I.—*Intemperance as a Disease.*

[We are indebted to Dr. Parrish, of Media, Penn., for the following *Report of the Committee on Intemperance as a Disease*, read before the State Medical Society of Pennsylvania at its recent session.—ED.]

REPORT.—The statement that there *is* a "medical aspect" to this subject, presupposes that intoxication, either from alcohol or opium, is, in some of its phases, a disease; and if this be true, it is an interesting inquiry how far this fact modifies the relation of the subject to morals and law.

In the very outset, we find ourselves confronted by recognized authorities in law and morals which, while they may not deny that intemperance is sometimes a disease, still deal with it exclusively as an offence against law and morality.

In this respect, it is an anomaly in our profession, because not only has it positive and well-defined symptoms by which even non-professional observers can distinguish it, but it is made a punishable offence against common decency and public order. Your committee therefore find it difficult to reconcile this peculiarity of the subject with the ordinary methods of professional thought and inquiry as related to other diseases which have no such embarrassments.

There are, however, distinct forms and varieties of alcoholic excess, which are well defined, and which we propose briefly to notice. Of that class of men who take their wine, as Cowper did his tea, "to cheer, but not to inebriate," we shall not speak. These are technically known as moderate drinkers, and are not necessarily subjects, on that account, for medical observation and treatment.

There are, however, at least three other classes:

1. The occasional drinker, who sometimes, but seldom, crosses the boundaries of sobriety.

2. The periodical drinker, who, at intervals, throws the reins on the neck of appetite, abandons himself to his passion for excitants, and who may do so almost unconsciously, or with deliberate intent and purpose.

3. The habitual drinker, who indulges without deliberation or without sudden impulse, but because there is an appetite which has corrupted his constitution, and made him a victim to a habit which he can not of himself overcome.

The multitudes of persons who are addicted to these several forms of inebriation, and the terrible consequences of the disease upon the domestic and industrial interests of the world, render its discussion one of the most important which can claim our thought. It seems to your committee to be a medical and physiological question, which should be studied as any other medical question; and in whatever legislation may be required concerning it, that this fact should be recognized.

*Causes.*—Your committee are further of the opinion that there are constitutional tendencies inherent in mankind to seek artificial support. In vindication of this statement, we find that in every soil and climate there is some indigenous product from which man, whatever be the stage of his civilization, extracts an intoxicating ingredient. The rudest people of the globe, without any of the appliances of modern art or science, are not so rude and uncultivated, that they do not manufacture alcoholic liquors from the growth of their fields; and we know of no nation or people on the earth among whom intoxication is not a common vice. There is a demand for intoxicants, and there is a supply.

Accepting this fact of history, comparing it with the experience of our own time, and noting the evidences which are daily presented to us in the practice of our profession, we are convinced, beyond doubt or controversy, that there does exist in men a tendency to the use of exhilarating beverages or narcotic drugs.

Man, frequently uneasy and restless, naturally longs for and seeks something to raise him from suffering, and elevate him to what he discovers to be a *pleasurable point* of feeling; a point which he may obtain from various sources, according to his tastes or inclinations. Sometimes it may be found in the pursuit of art, science, literature, or society; and frequently in the productions of the animal and vegetable world.

The prudent man, especially if he be a man of domestic habits, may be content with the exhilaration afforded by his tea or coffee, and his cigar. One may be satisfied with home-made wine, cider, or beer; another thinks he requires a stronger stimulant, and another a still stronger, who partakes of more dangerous varieties, till he becomes narcotized or drunken.

Whisky and brandy are the intoxicants of America, Russia, Scotland, and Ireland; ale and beer, of England, Germany, Japan, and Egypt; wine, of France and Italy; bouza, of Nubia; pulque, of Mexico; tuka, of Kamschatka; betil, of Polynesia; arrack, of Africa and Hindoostan; opium and shamshu of China and Turkey; bangue and hashish, of Arabia and the Grecian Archipelago; coca leaves, of Peru; palm leaves, of the palm countries; hyoscyamus, of Syria; rue, of the Crimean valley; and, in more recent times, ether and chloroform among the cultivated and refined of our own country.

It thus appears clear that the production of intoxicating beverages or drugs is restricted to no country or clime. It is as widely spread as the existence of language. Wherever we find a national speech, there we as certainly find a national intoxicant; nor does this remark apply only to the present age, but, as far as the voice of history can be heard, it applies to *every* age of the world. Whence this universality of production? How happens it that, in every country and clime, plants grow, which are capable of yielding intoxicating products — such products as possess the threefold quality of exhilarant, roborant, and anodyne, and which are either deleterious or beneficial according to the quantity used? Observe the adaptation. Man, says a poet and philosopher, is at once "*the child and sure heir of pain.*" He needs many a time such a draught as may exhilarate, strengthen, or compose. Nature, aided by human ingenuity, supplies it. Shall we say that man must not partake of these products? Let us consider. Nature offers them with a liberal hand. There is within man a sense of want, which bids him partake. Nature, however, human experience, our own observation, and the moral law, prescribe the limit — *moderation;* beyond which, suffering is the result.

In view of the wide-spread evils resulting from the careless and indiscriminate use of such beverages, it seems to be the province of the medical profession to recommend only the common use of such as experience has proved to be safe, and abstinence from those known to be injurious.

A philosophic writer has said: "*Every inordinate cup is unblest, and the ingredient is a devil.*"

Unhappily, men do not partake in moderation, and therefore must, and do, endure the penalty. With a "demon" in the breast, their sufferings are fearful, till even *death* seems a shelter from the agony of a lacerated constitution and an avenging conscience. In this category falls the habitual drunkard.

What is our duty towards him? What prescription has the medical fraternity that shall meet his case? Are there any means even to restore partially our brother man to ease and serenity, and to throw around his spirit a shadow of hope even for corporal salvation? We believe there are such means.

The popular cry is, "Remove the cause." If we ask for the cause, the answer is, "The dramshops, and the law by which they are authorized and sustained." In view, however, of the momentous facts already referred to, your committee must take a broader and deeper view of the causes which underlie this evil. It is alike unphilosophical and unjust to classify social usages and dramshops as the chief causes of intemperance. They are temptations, *not* causes; temptations which are in the way of sober people as well as of those who drink. In dealing with this subject, we should be careful in the use of terms, and understand the terms we use.

A cause is an *invariable antecedent.* Drinking at dramshops or in social circles does not invariably antecede the habit of drunkenness, but a susceptibility or capacity for such a condition must always exist in the person who becomes an inebriate. The dramshop or social glass may only be the occasion.

Some forms of intoxication are peculiarly exclusive. The custom of "treating" at public bars is applicable only to a class. The habitual drinker indulges at home, in his office or place of business, at particular times and under circumstances of unusual effort or exposure. He does not think of depending upon invitations and associations for the enjoyment of his accustomed draught. With him the demand is from within; a craving analogous to that of hunger, which he feels must be satisfied or he is unfitted for the service of life.

Many a periodical inebriate carries liquor to his room, and remains for days, and even weeks, in seclusion, while he passes through the satisfying narcotism which obliterates all thought and care from his mind, and puts his body in a condition of protracted slumber and repose.

Opium-using is a secret vice. Hashish is used privately, and ether and chloroform are inhaled in solitary places.

In considering this matter, it is well also to bear in mind the fact that the majority of persons who are exposed to these temptations do not become inebriates. In other words, they are either free from the desire for such indulgence, or they have moral power sufficient to enable them to subdue and control their appetites. Only those people become drunken who possess the susceptibilities and tendencies which incline them to seek excitants, and who, on that account, are more likely to yield to temptation. Why do some men drink to excess? Why do others, who are exposed to the same temptations, abstain from drinking at all? When we reach the difference between these two classes of persons, we reach causes.

It is therefore respectfully submitted that if intemperance is an offence, an immorality, the distinction is unfair which reproaches the man who establishes the habit of intoxication by the use of alcoholic liquors, while he who does the same thing by the use of opium or other drug is relieved of such reproach.

When we consider the damaging influence of the opium habit especially, how it degenerates the nervous force, demoralizes manhood, and paralyzes labor—how it holds its victim a bondman, with no intermission of freedom and no promise of escape—it is difficult to comprehend the ethical code which passes such slavery by as innocent, and yet holds the less serious case as guilty.

The singular compounding of these diseased conditions with judicial opprobrium and public disgrace, is a point which we feel called upon to notice.

*Civil Aspect.*—This brings us to the civil aspect of the subject. The time was when insane persons were punished; they were victims of an unrighteous law and a bitter public sentiment. They were chained, beaten, and starved, and the madhouses were Bedlams where the hard hand of tyranny crushed the sad victims of mental disorder. Nor was it until the benign attributes of the medical profession were brought to bear upon the subject of insanity, that its treatment was transferred from the prison-keeper to the doctor of medicine.

What was true of the insane a century ago, is, with some modification, true of the drunkard now. The law regards him as an offender, subjects him to arrest and fine, or imprisonment, and the public looks upon him as a nuisance and burden. His sorrows are made texts for sermons; his eccentricities are caricatured by itinerant lecturers; his bruises and scars are pictured for public stereoscopic displays; and the tendency of the popular teaching of the day is to separate him from public sympathy, and oppress him with the sense of a self-imposed and willing degradation. We admit that he has committed an offence, but that he is not on that account to be deprived of sympathy and assistance.

Physicians, whose calling leads them among scenes of distress, and whose counsel and confidence are sought to relieve those who suffer, are accustomed to draw a veil between the scars and tears of families where they minister, and the gaze of the world. This being their sacred office, they look with detestation upon the common practice of making a display of the drunkard and his household for public entertainment, and are free to express their unqualified opposition to such a course.

*Duty of Physicians.* — We have now reached the inquiry as to the duty of physicians, and would first remark upon our relation to the inebriate in private practice. At present, the drinking man hesitates to consult his physician till the occurrence of mania or other acute symptoms makes it necessary. He realizes that he is disgraced, and hesitates to relate his symptoms to any one. His honor and manhood being compromised, he makes a determined effort to maintain his independence, and avoids conversation or counsel about himself. He thinks there is no sympathy for him, and, brooding over the compunctions and sorrows that are within him, abandons himself to his forlorn condition, and bears alone the burden that should be shared with his medical adviser.

This estrangement between physician and patient doubtless has its origin in the idea of criminality that is associated with the fact of intemperance; and it is only needful that it should be publicly recognized as a disease, in order to secure confidence and freedom on both sides. Until this is done, there can be no reasonable basis for professional counsel.

On the part of physicians themselves, there does not seem to be a full appreciation of their position and its requirements. Let us consider this a moment. It is said that we, as a profession, are doing much to promote intemperance by the administration of alcoholic remedies. Whether this be true or not, is a question which each one of us should gravely consider for himself. We have all the varieties of intoxicating beverages, and more than two hundred alcoholic remedies, at our command, for the treatment of all sorts of diseases requiring stimulants and narcotics, and it would be unreasonable, and even arrogant, to suppose that there is not occasionally misdirection in their choice and prescription. All this may be acknowledged, without admitting any more than the fallibility of human judgment and the uncertainty of human skill. While we have no sympathy with the wholesale clamor against the profession on this account, we believe there is need of caution in the administration of intoxicants to persons in whom the tendency to excess in their use is apparent.

The relation of our profession to this subject is peculiar, and, for this reason, important and responsible. No other class of men has a legal right to authorize persons to use intoxicants. Ministers of religion may teach the principles of temperance; lawyers may stand in the breach between the law and the offender; lecturers may be very eloquent over the evils of excess; but physicians only are authorized to *prescribe and direct the use* of these articles. So far as the consumer is concerned, he occupies a very different position when he takes them from the hand of his medical adviser, and when he goes of his own accord to a legalized place of sale and purchases them. In the one case he relieves himself of responsibility, and in the other he assumes it. To him the moral aspect of the case is widely different.

What, then, is required of us? In considering this question, we are impressed, first, with the conviction that it is our duty to influence the public mind, so far as we may, in the direction of sympathy for the *entire* class of excessive drinkers. Intemperance is a public evil, and, as such, we must reach it primarily through public sentiment. That sentiment should lay aside its reproach and disgust, and adorn itself with charity. When this shall be done, society will have taken its first step towards the recovery of the inebriate, and its own security.

The method which is now popularly employed is to appeal to the conscience of the inebriate. His conscience admits the reasonableness and justice of the appeal. He knows, but does not perform. Like Paul, the apostle, he declares: "For to will is present with me, but how to perform that which is good I find not." A distinction is thus drawn between willing to do a thing, and the power to do it; and because this distinction is not incorporated with the morality which is popularly applied to this subject, the efforts to reform inebriates by such appeals are usually unsuccessful. Conscience, in such cases, is sensitive — so sensitive that it shrinks, like a hurt child, from contact even with what is good for it — but its power is gone. It is a slave to passion. It strives with the gross and hurtful, but yet it is overcome. As a philosopher has substantially said: If conscience were as powerful to do as it is sensitive to feel, and authoritative to direct, the world would soon find itself on the side of right.

In addition to this, a subscription to a pledge of total abstinence is demanded, and that it may prove effectual, the influences of association, and all the dramatic effect of secret and imposing rituals is added; but these reach only exceptional cases. The result of all this kind of effort, sustained by so much talent and piety, and continned so perseveringly over so many years, and sometimes incorporated with political action, has not, in the opinion of your committee, accomplished what its friends and supporters had reason to anticipate. We have no controversy with such efforts, nor do we discredit their sincerity and earnestness, but simply deplore their failure to accomplish the good which was intended.

As physicians, we have specific duties to perform. There is reason to fear that alcoholic liquors are employed too freely, especially among young persons; and that such practice is productive of evil consequences. We should see to it that we do nothing that may prove the occasion for excess on the part of those who commit themselves to our care, if it can be avoided. We are also called upon to instruct the families for whom we prescribe, and the communities in which we live, concerning both the causes of intemperance, and the uses of alcohol. Popular ethics classes all beverages containing alcohol among the poisons, and popular ignorance raises its voice against their use on this account. Notwithstanding this, one truth still exists, viz.: that nature, science and experience can not be obliterated by the clamorous platitudes of men who, however good their intentions may be, are not the competent tribunal by whom this question of the use of alcohol is to be determined. Nature is the founder, science the expositor, and experience the judge, by whom we are to be guided in the decision of this question.

We have seen that the experience of mankind the world over testifies to, and defends the use of such excitants for the purpose of exhilaration and enjoyment. Let us now notice the never-to-be-forgotten fact, that they are also used for the purpose of supporting the system in the absence of ordinary food. That this is the case is one of the most astonishing results of human discovery. Anstie reports well-authenticated and extraordinary instances of the power of alcoholic liquors not only to sustain life in disease, but to supplement food in conditions of health; and it is probable that such instances have occurred in the experience of most of the members of this Society. He speaks of alcohol as an article of diet, and you may remember the case of the octogenarian soldier, who had subsisted for twenty years, in good health, on a bottle of gin, and a small crust of bread daily. It is a well-known fact, that the coca chewers of Peru are capable of sustaining a vast amount of labor for a very long time without food, if they can be allowed their accustomed quantity of coca. The power of tobacco, also, to compensate the want of ordinary food, is too well known, not only by consumers of the weed, but by all well-informed persons, to need demonstration. Soldiers, during our late war, frequently and cheerfully sacrificed a ration for the sake of a "quid."

Opium is remarkable for this compensating property. Eastern travelers have publicly declared, that their horsemen and guides would do more work under the stimulation of moderate quantities of opium, than by taking the habitual meal without it. The workmen in opium factories enjoy as good health as those who work in other factories. According to De Quincey, the operatives in the mills of Manchester use the drug, not to produce exhilaration, but to relieve what we have referred to in this report, as an uneasy, restless feeling, but what he calls a sense of fatigue and depression. It is for this purpose it is commonly used among the Orientals; but as its repetition is necessary to keep up the impression, the habit gradually fixes itself in the system.

Objection is made to the use of alcohol, because its effect is to stimulate temporarily, and it requires repetition. We submit that this objection does not benefit the argument against the use of alcohol. The same may be said of the food we eat, and the air we breathe. We constantly need the punctually returning meal, and the ever present vital influence of atmospheric air. The suspension of either would be fatal.

It is assumed, also, that alcohol is not transformed in the system. The same may be said of water, without which we cannot survive. It is water all the time, and escapes in the same quantity that it is received into the body. In admitting this assumption (that alcohol is not transformed) for the sake of argument, your Committee do not adopt it as true, because the most reliable observers and experimenters upon the subject have demonstrated that alcohol may be appropriated by the system, and sustain life in the same way that food is appropriated, and enters into the nutrition of the body. Close observation in the alcoholic treatment of several diseases will frequently discover this fact. Large quantities may be taken by the patient, and there is neither exhalation of alcoholic odor from the lungs, or trace of it in any of the secretions.

We do not present these facts as arguments in favor of the habitual use of alcohol or any other inebriant, but simply as physiological truths which we cannot gainsay. It is bad logic and philosophy, and *worse* than bad physiology and therapeutics, to object to the use of alcohol on the ground that it is a poison, or that it is not assimilated.

In urging upon the non-medical advocates of reform to award to alcohol its true place and power, we are but fortifying them with truth, without which no cause will prosper. There are natural and prudential reasons why alcohol should be used with the utmost caution, and there is no need of straining truth, or perverting scientific facts in order to find arguments in favor of temperance.

*Relation of the State.*—The next point to which we invite attention is the relation of the State to this subject, and, in discussing it, we may simply advert to her position towards others who claim her care. It is taken for granted, by all classes, that the number of habitual inebriates in Pennsylvania, is much greater than all the insane, blind, deaf-mutes, etc., for whom the State makes liberal provision, and it is assumed by sociologists, and heralded throughout the land, that intemperance is one of the chief causes of these several maladies; yet, while we have institutions reared by public and private benefactions for all of them, neither the wisdom of our legislators, nor the philanthropy of our people has comprehended the necessity of offering shelter, and furnishing judicious care for a class of persons, of whom there are none more cultivated in intellect, more generous in heart, or more capable, under conditions of health, of adding to the culture and prosperity of the Commonwealth.

The question may be asked, What experience have we to justify the establishment of institutions for the care and treatment of persons who are addicted to alcoholic and opium excess? Our answer is that the experiment has been successfully tried in Massachusetts and New York, under the patronage of these States, and, for the last two years, at Media, Pennsylvania, by a private corporation. The results have been more satisfactory than with any other class of hospital practice, excepting only accidents and acute diseases. Your Committee believe it is not claimed by any of the distinguished gentlemen who have charge of hospitals for the insane in this country that even twenty per cent. of chronic cases recover; and chronic cases are generally understood to be those whose insanity has existed more than a year. Patients, however, who have been addicted to excess in the use of alcohol, opium, etc., from five to twenty years, have recovered in a larger proportion than is claimed for chronic insanity, though such persons, if they would continue free from danger, should totally abstain from intoxicants.

Another question is not unfrequently asked, to wit: Are the cures permanent? From ignorant or unprofessional minds this question may be excusable, but from physicians it is quite the reverse. It is a question the propriety of which we would not acknowledge, as related to any other known disease. We attend patients with fever, rheumatism, pneumonia, etc., and they recover; but no conscientious and intelligent practitioner will say "the cure is permanent," and advise his patient to go into the world again, pursue his business, and expose himself to all the influences which induced his attack, with the assurance that he will never have a recurrence of the disease. We send our patients to hospitals for the insane, and to general hospitals, according to their necessities; but the physicians in charge of such institutions do not send them back to us cured, and tell us "the cure is permanent." Such a statement would imply a re-creation of the patient without the tendencies and infirmities common to men.

The testimony of those whose experience in this matter entitles their opinion to consideration and influence, is that from thirty to fifty per cent. of patients in institutions for the intemperate, who were formerly a burden to their families, return again to society, capable of successful occupation in the affairs of life. That this percentage is large and satisfactory all will admit, and that it may be increased with advanced knowledge and improved appliances, there can be no reasonable doubt.

*Are Inebriates Insane?*—We have now reached a point, in this discussion, of great practical moment to those who are addicted to alcoholic and opium excess. Are such persons insane? To answer this question logically and fairly, we must distinguish.

The word insane, according to its etymology, means unsound, and in this indeterminate sense is often loosely used. If this broad definition be accepted, every man who exhibits disordered mental action, is insane. Under it is embraced not merely the drunkard, but all human beings. To use the word in this sense, therefore, would be manifestly unreasonable. Few persons would be willing to hazard the opinion that the celebrated Thomas De Quincey and Samuel Coleridge were insane, even through the years of intemperate indulgence during which their celebrity was chiefly gained. Men who are intemperate, either from opium or brandy, are not, in the majority of cases, men of insane intellect. Medical observation and diagnosis have, we think, distinctly proved that the diseased portion of the mind in such cases is chiefly of the will, not the intellect. They know, but are *impotent to perform.* An able medical writer, Dr. John Reid, in speaking of nervous disorders, says: "We often act upon the ill-founded idea that such complaints are altogether dependent upon the power of the will, a notion which, in paradoxical extravagance, scarcely yields to the doctrine that no one need die, if with sufficient energy he determined to live."

An intoxicated man may have hallucinations, be troublesome, and even violent, but such irregularities are analogous to symptoms of mental disturbance that are frequently witnessed in the course of acute disease — as the delirium in fever, and in the various forms of cerebral inflammation — and which passes off in a few hours or days, without the patient being considered insane.

Hallucinations and illusions may exist without insanity. They do not necessarily involve perversion of intellect or judgment. Indeed the reason may be quite clear, and competent to discover the existence and causes of those sensations, without being able to control them. Writers upon this subject speak of "insanity of the will," by which they mean a *perverted* will, that prompts to extraordinary acts which the insane person commits with full intent, and matured design, and enjoys satisfaction with the result, however distressing or dreadful it may be to others. Many inebriates *will* to abstain from excess in the use of intoxicants, and, indeed, determine to abstain totally, until the occasion presents which controls the will, but does not pervert it.

They act in opposition to it. They are captives, and the will yields to the insatiable demand of physical unrest and depression, or moral infirmity. When it is over, they are stung with the bitterest remorse, and sink into the deepest penitence and sorrow. Such, however, are not the fruits of insanity.

If these propositions are true, it is clear that institutions for insane persons are not the best means for the restoration of the class we are considering, and we think we have the concurrent judgment of most, if not all, *experts* in the department of insanity, in support of this opinion.

*The Opium Habit.*—In conclusion, your Committee beg leave to refer briefly to a single fact concerning opium, without which this report would not cover the whole subject referred to the Committee.

The opinion that the opium habit is greatly on the increase in nearly all communities seems to be gaining strength, and especially does it seem to be increasing in those communities and states where the sale of intoxicating liquors is prohibited by law.

It is generally admitted that opium and alcohol are more largely used in medicine than any other drugs, and it cannot be denied that the effects of the abuse of these two wonderful products are most pernicious and destructive. They cannot be abandoned as remedies. They are too essential and too potent to be overlooked. They come to us laden with power for good, when properly used, and power for evil, when abused. Increased care on our part, increased intelligence concerning their true qualities and place, on the part of the people; and a sounder philosophy on the whole question of human necessities, by public teachers, will do much to correct the evils from which the community now suffers.

We have stated that from thirty to fifty per cent. of cases of habitual alcoholic intoxication are curable, and we now make the additional statement, that from our own observation, ninety per cent. of opium cases are curable, under institution treatment.

Your Committee regret that they have not been able to present a more complete and exhaustive report; but with very little literature and still less authority on the subject, we have been compelled to draw upon our own limited observation and experience for most of our propositions and conclusions; and we ask for them a candid review and criticism by the profession, whose responsible and dignified prerogative as teachers and reformers has been frankly set forth in the body of this report.

JOSEPH PARRISH,
EDWARD WALLACE,
WM. B. ATKINSON,
JAMES KING,
JACOB PRICE,
*Committee.*

